# Complexes of D-type cyclins with CDKs during maize germination

**DOI:** 10.1093/jxb/ert340

**Published:** 2013-10-14

**Authors:** Silvia K. Godínez-Palma, Elpidio García, María de la Paz Sánchez, Fernando Rosas, Jorge M. Vázquez-Ramos

**Affiliations:** ^1^Facultad de Química, Departamento de Bioquímica, UNAM, Avenida Universidad y Copilco, México DF 04510, México; ^2^Instituto de Ecología, UNAM, Avenida Universidad y Copilco, México DF 04510, México

**Keywords:** CDKA, CDKB, cell cycle, D-type cyclins, germination, maize.

## Abstract

The importance of cell proliferation in plant growth and development has been well documented. The majority of studies on basic cell cycle mechanisms in plants have been at the level of gene expression and much less knowledge has accumulated in terms of protein interactions and activation. Two key proteins, cyclins and cyclin-dependent kinases (CDKs) are fundamental for cell cycle regulation and advancement. Our aim has been to understand the role of D-type cyclins and type A and B CDKs in the cell cycle taking place during a developmental process such as maize seed germination. Results indicate that three maize D-type cyclins—D2;2, D4;2, and D5;3—(G_1_-S cyclins by definition) bind and activate two different types of CDK—A and B1;1—in a differential way during germination. Whereas CDKA–D-type cyclin complexes are more active at early germination times than at later times, it was surprising to observe that CDKB1;1, a supposedly G_2_-M kinase, bound in a differential way to all D-type cyclins tested during germination. Binding to cyclin D2;2 was detectable at all germination times, forming a complex with kinase activity, whereas binding to D4;2 and D5;3 was more variable; in particular, D5;3 was only detected at late germination times. Results are discussed in terms of cell cycle advancement and its importance for seed germination.

## Introduction

The cell cycle has a preponderant role in the growth and development of multicellular organisms, including plants (Dewitte and Murray, 2003). The basic cell cycle mechanisms are similar between plant and mammal cells, showing a high conservation throughout evolution. Nonetheless, some differences exist, particularly regarding regulatory processes and the amount of proteins involved, perhaps reflecting strategic differences in plant life.

Similar to other eukaryotes, in plant cells there are cyclin-dependent kinases (CDKs) that, in association with a cyclin, determine cell cycle progression. D-type cyclin–CDK complexes control G_1_–S-phase transition due to their capacity to interact with the retinoblastoma-related (RBR) protein. This is mediated by the presence of the LXCXE sequence in cyclins and the subsequent phosphorylation of serine and threonine residues located in the carboxy end of RBR by the CDK moiety. This provokes dissociation between RBR and the transcriptional factor E2F/DP, thus allowing transcription of those genes necessary for S-phase establishment (Renaudin *et al.*, 1996).

Cyclins, as their name suggests, fluctuate throughout the cell cycle (Evans *et al.*, 1983), in this way regulating CDK activity; moreover, cyclins are necessary for CDK activation, thus modifying the stability, location, and substrate specificity of the CDK–cyclin complex (Inzé and De Veylder 2006). Cyclin stability is mediated by proteasome degradation (Tyers and Jorgensen 2000; Fyvie *et al.*, 2002), and CDK activity depends on its phosphorylation/dephosphorylation state (Riou-Khamlichi *et al.*, 1999) and its interaction with protein kinase inhibitors (Verkest *et al*., 2005*a*, 2005*b*).

Cyclins D act as sensors of external stimuli so their presence is a fundamental regulatory factor for cell cycle initiation (Cross 1988, 1990; Nash *et al.*, 1988; Hadwiger *et al.*, 1989; Hadwiger and Reed, 1990). Depending on their destruction mechanism, these cyclins can be constitutively unstable during the cell cycle or unstable only in certain stages of the cell cycle (Pines 1995). The degradation pathway of D-type cyclins in plants is still to be clearly defined; however, there is evidence for the action of the ubiquitin-proteasome system and in fact most D-type cyclins possess PEST sequences in their protein sequence.

The cell cycle plays a very important role in seed germination (Vázquez-Ramos and Sánchez, 2003). Seed germination is a physiological process that starts with water entry (imbibition) and it is thought to end when the radicle protrudes (Bewley, 1997). Some reports indicate that radicle protrusion does not require mitotic activity (Baiza *et al.*, 1989), while others demonstrate that cell division takes place before protrusion (De Castro *et al*., 2000; Barroco *et al.*, 2005; Masubelele *et al.*, 2005). The cell cycle stage of most cells in dry seed embryos seems to be G_1_ phase (Deltour and Jacqmard, 1975; Conger and Carabia, 1976; Bewley and Black, 1994), and many different cell cycle proteins are already present in non-imbibed embryos, suggesting that cells are prepared to start the cycle (Bray *et al.*, 1989; Bewley, 1997; Vázquez-Ramos and Sánchez, 2003); *de novo* synthesis of cell cycle proteins appears to start some hours after imbibition. Thus, in maize, DNA replication starts by 12–15h of imbibition, as determined by ^3^H-thymidine incorporation, nuclear labelling, histone H1 biosynthesis, proliferating cell nuclear antigen (PCNA) accumulation, and DNA polymerase, DNA ligase, and DNA primase activities (Baiza *et al.*, 1989; Georgieva *et al.*, 1994*a*, 1994*b*; García *et al.*, 1997; Vázquez-Ramos and Sánchez, 2003). Mitotic figures are evident after 24–28h of imbibition (Baiza *et al.*, 1989).

During seed germination D-type cyclins play essential roles, as indicated by work with homozygous mutants for the cyclin D1 and D4;1 genes in *Arabidopsis* that show a dramatic delay in cell division and proliferation during seed germination (Masubelele *et al.*, 2005), with a corresponding delay in radicle protrusion.

Plant cells have multiple D-type cyclins but only some of them have been studied and limited data exist on their interaction with CDKs and their function, particularly during seed germination. Maize cells contain 17 different genes coding for D-type cyclins, 15 of which are expressed during germination (Buendía-Monreal *et al.*, 2011); three of them have also been studied at the protein level (Gutiérrez *et al.*, 2005; Lara-Núñez *et al.*, 2008). The aim of this work was to study complexes formed by three maize cyclins—CycD2;2 (a or b, 94% identical), CycD4;2, and CycD5;3 (a or b, 74% identical) with CDKs (types A and B1;1) during the early stages of maize germination, following D-type cyclin protein levels, the type of complexes formed between different cyclins and CDKs, and their activity during maize germination.

## Materials and methods

### Materials

Protein A–agarose and protease inhibitor cocktail tablets (Complete), were from Roche (Indianapolis, IN, USA). Western chemiluminescent horseradish peroxidase substrate kit and Immobilon polyvinylidene fluoride membranes were from Millipore (Billerica, MA, USA); glutathione–Sepharose 4B and plasmid pGEX-4T2 were from Amersham Biosciences (Little Chalfont, Bucks, UK); anti-rabbit IgG–horseradish peroxidase conjugate was from Santa Cruz Biotechnology (sc-2004; Santa Cruz, CA, USA); [γ-^32^P]ATP was from Isotopes Co (Budapest, Hungary); reduced glutathione, calf intestinal alkaline phosphatase (1382074), and plasmid pPROEX-HTB were from Invitrogen, Life Technologies (Carlsbad, CA, USA); and phosphatase inhibitor cocktail 2 was from Sigma-Aldrich (P5726; St Louis, MO, USA).

### Imbibition of maize embryo axes

To follow germination (time from onset of imbibition), maize (open pollination genotype cv. Chalqueño) embryo axes (25–30mg dry weight) were washed four times with sterile distilled water. Excess water was removed by blotting the axes with sterile Whatman paper No. 1 and then incubated for 6, 12, 18, and 24h at 25 °C between sterile Whatman paper No. 1 with sterile imbibition buffer containing 50mM KCl, 10mM MgCl_2_, 50mM Tris/HCl, pH 7.6, 2% sucrose, and 10mg ml^−1^ chloramphenicol.

### Protein extraction

After maize embryo axis imbibition, protein extracts were produced by grinding axes in liquid nitrogen in a mortar and then adding extraction buffer containing 25mM Tris/HCl, pH 7.5, 15mM MgCl_2_, 75mM NaCl, 25mM KCl, 5mM EDTA, pH 8.0, 1mM dithiothreitol, 0.2% Triton X-100, 0.25M sucrose, 60mM β-glycerol phosphate, 50mM NaF, 200 µM Na_3_VO_4_, 1mM EGTA, a tablet of protease inhibitor cocktail/15ml buffer, and a tablet of phosphatase inhibitor cocktail. Protein extracts were centrifuged at 16000 *g* for 1h at 4 °C and protein concentration was determined by the method of Bradford (1976).

### Polyclonal antibody production

Rabbits were injected intraperitoneally with purified glutathione S-transferase (GST)–CycD4;2 (33kDa, 250 µg) or GST–CycD5;3 (37kDa, 250 µg) recombinant proteins, containing the carboxyl ends of CycD4;2 (amino acids 313–388) and CycD5;3a (amino acids 249–354; sharing strong identity with CycD5;3b in this polypeptide region). For CDKB1;1, a peptide containing the first 28 amino acids fused to GST was used (28kDa, 250 µg). The complete CDKA polypeptide (37kDa, 250 µg), fused to a His-tag, was used to raise antibodies. For the first injection recombinant proteins were mixed with complete Freund’s adjuvant (Sigma-Aldrich); a second injection contained only incomplete adjuvant. Further injections (weekly for 2 months) were administered through the popliteal ganglion with only the cyclin peptides (200 µg; purified by treating fusion proteins with thrombin protease and then passing the mixture through glutathione–Sepharose 4B to eliminate GST), the complete His-CDKA polypeptide (200 µg), or GST–CDKB1;1 peptide (200 µg). At the end of this period the antisera raised were collected and evaluated for their ability to detect the corresponding proteins. Antibodies against CycD2;2 were reported by Gutiérrez *et al*. (2005).

### Western blotting

Protein samples (50 µg) were fractionated by SDS/PAGE (12%) and gels were blotted onto polyvinylidene fluoride membranes. Membranes were blocked with a 3:10 dilution of fetal bovine serum/PBS 1× (SO1520-biowest) and then incubated with one of these polyclonal antibodies, developed in our laboratory: anti-maize CycD2;2 (1:1000 dilution), anti-maize CycD5;3 (1:2500 dilution), anti-maize CycD4;2 (1:1000 dilution), anti-maize CDKA (1:1000 dilution), or anti-maize CDKB1;1 (1:1000 dilution). They were incubated overnight at 4 °C and washed three times with PBS, 0.5M NaCl, and 1% Triton X-100 for 15min. Subsequently, membranes were incubated for 1h with peroxidase-conjugated anti-rabbit antibody at a 1:40000 dilution. Membranes were washed again three times with PBS, 0.5M NaCl, and 1% Triton X-100 for 15min each. Peroxidase reaction was detected by the ECL method. Densitometric analysis was performed using the Fluor-S MultiImager (Bio-Rad, Hercules, CA, USA). Anti-CDKA antibody strongly recognizes CDKA, and it also recognizes CDKB1;1, albeit weakly. Anti-CDKB1;1 antibody only recognizes CDKB1;1. Assays to demonstrate that the anti-CDKA antibody recognizes CDKB1;1 required protein concentrations above 200 µg (Fig. S2E).

### Immunoprecipitation

Anti-CycD4;2, anti-CycD5;3, anti-CycD2;2, anti-CDKA, or anti-CDKB1;1 antibodies were conjugated for 2h with protein A–agarose (6:15 dilution) at room temperature using buffer A (25mM Tris/HCl, pH 7.5, 125mM NaCl, 2.5mM EDTA, pH 8.0, 2.5mM EGTA, 2.5mM NaF, and 0.1% Triton X-100). Protein from extracts (150 µg) was added and the mixture was incubated overnight at 4 °C with agitation (for experiments of antibody cross-reactivity for CDKA and CDKB1;1, protein concentrations were 100, 150, 200, and 300 µg; Fig. S2F), immunocomplexes were pelleted by centrifugation in a microfuge and washed three times with buffer A. Subsequently, antibodies against maize CDKA (1:1000 dilution) or against maize CDKB1;1 (1:1000 dilution) were added to identify the corresponding CDK by western blotting. The resulting protein precipitates were used as the source of kinase activity. The target protein was identified after every immunoprecipitation. For dephosphorylation assays, immunoprecipitates were incubated for 40min at 36 °C with alkaline phosphatase (5U); then phosphatase inhibitor (5:1 ratio) was added and the mixture was incubated 40min at 36 °C. Immunoprecipitates were washed three times with Tris/HCl buffer (pH 7.5) and three times with buffer A, and then kinase activity was measured.

### Kinase assay

Immunocomplexes were incubated in 10 μl of kinase buffer (70mM Tris/HCl, pH 7.5, 10mM MgCl_2_, 150mM NaCl, 1mM dithiothreitol, 5mM EGTA, 20mM ATP, and 5 μCi [γ-^32^P]ATP). As substrate, GST–Zm-RBR-C fusion peptide (RBR-C is the C-terminal domain of maize RBR) was added at 5 µg per sample. Purification of GST–RBR fusion peptide was performed according to Ramírez-Parra *et al*. (1999). Reactions were performed for 1h at 30 °C and these were stopped by adding SDS loading buffer. After boiling for 5min, the reaction products were separated by SDS/PAGE. The gels were vacuum-dried at 80 °C for 2h and then were exposed for 12h to Hyperfilm ECL (Amersham Biosciences). GBX developer and GBX fixer (Kodak, Rochester, NY, USA) were used to develop films.

### Sequential immunoprecipitations

Initial immunoprecipitations were performed using anti-Cyclin D2;2, D4;2, or D5;3 antibodies, protein A–agarose, and 150 μg of protein per germination time; immunoprecipitates were washed and subjected to treatment at 65 °C for 3h to separate cyclin–CDK complexes from protein A–agarose. Then they were centrifuged at 5000rpm for 10min and supernatants were incubated for 12h with anti-CDKB1;1 antibody, and then centrifuged again at 5000rpm for 5min and supernatants were immunoprecipitated with anti-CDKA antibody. Kinase activity was determined in pellets resulting from immunoprecipitation with both anti-CDKB1;1 and anti-CDKA antibodies.

## Results

### Specificity of antibodies against maize D-type cyclins and CDKs

Antibodies are fundamental tools with which to test the presence and associations of proteins in the cell. For this purpose, specific peptide sequences of maize cyclins D4;2 and D5;3, and CDKB1;1 and the complete protein sequence of CDKA, were purified and used to produce the corresponding polyclonal antibodies. Anti-CycD2;2 antibodies have been reported before (Gutiérrez *et al.*, 2005).

**Fig. 1.**
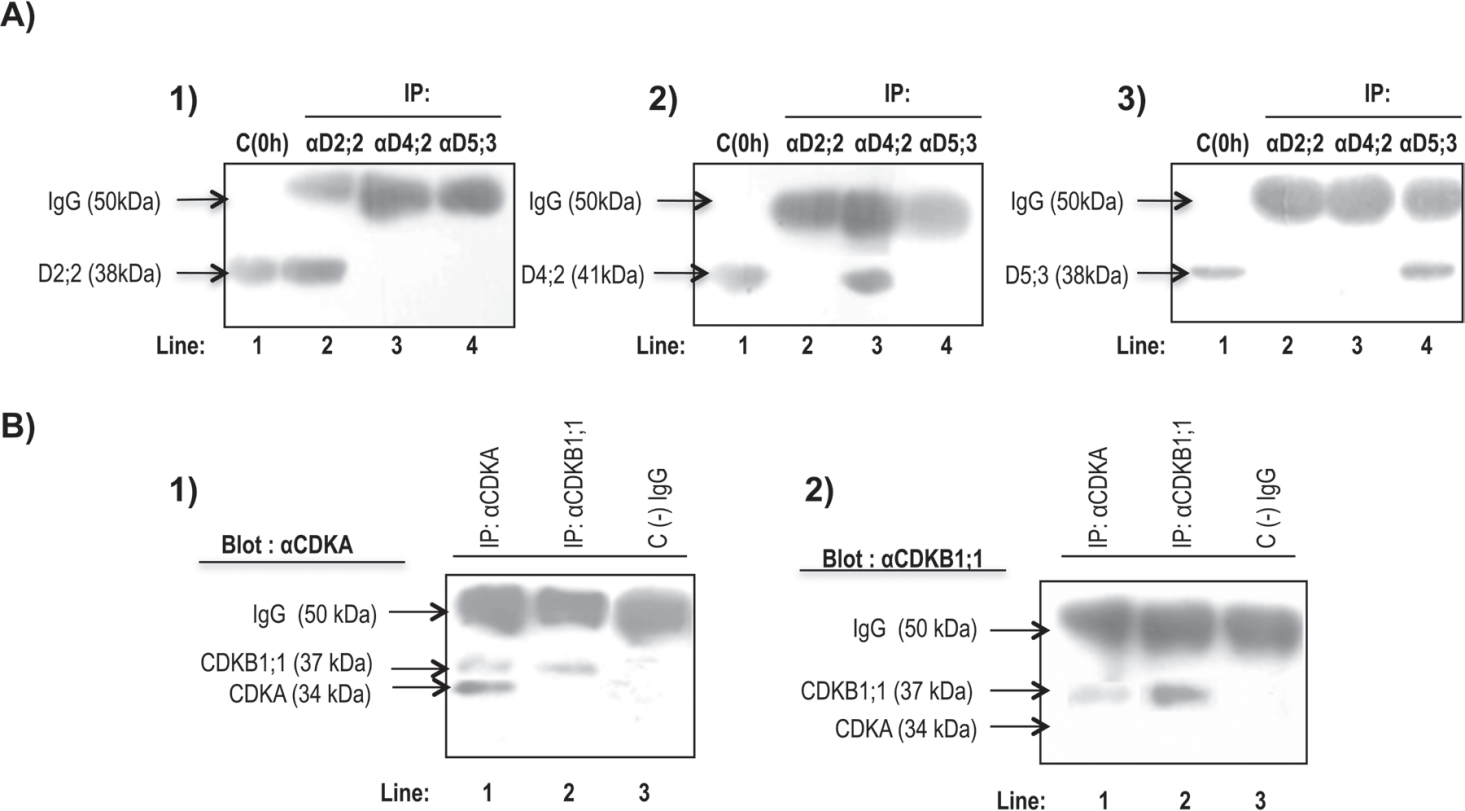
Specificity of polyclonal antibodies. (A) Western blots using antibodies against the corresponding cyclins D: lanes 1, protein extracts from unimbibed maize axes; lanes 2–4, immunoprecipitation with anti-CycD2;2, anti-CycD4;2, and anti-CycD5;3 antibodies, respectively. Panels 1–3 show western blots using anti-CycD2;2, anti-CycD4;2, and anti-CycD5;3 antibodies, respectively. (B) Specificity of anti-CDK antibodies; lanes 1, immunoprecipitation with anti-CDKA antibody; lanes 2, immunoprecipitation with anti-CDKB1;1 antibody; lanes 3, immunoprecipitation with anti-CDKB1;1 antibody, but without adding protein extracts, showing only high-molecular-weight IgGs. Panels 1 and 2 show western blots using anti-CDKA and anti-CDKB1;1 antibodies, respectively.

The D-type cyclin peptide sequences used were such that percentage identity between cyclins was always below 30% (BioEdit v7.2.0; Hall, 1999; Fig. S1A). Thus, no cross-reactivity was observed with the different antibodies (Fig. 1A and Fig. S2A, C). However, the corresponding antibodies cannot differentiate two very closely related cyclins D2;2 (a and b) and two closely related cyclins D5;3 (a and b).

The anti-CDKA antibody, produced against the complete protein, can recognize both CDKA and, to a lesser extent, CDKB1;1 (protein concentration above 200 µg is required), since both proteins share high identity in their sequences, with the exception of the amino tail in CDKB1;1, which is specific and not present in CDKA proteins (Fig. S1B). The anti-CDKB1;1 antibody was produced using only the CDKB1;1 amino tail (Fig. 1B and Fig. S2B, D, E, and F). Immunoprecipitations using anti-CDKA antibody show bands of 34 and 37kDa (Fig. 1B, panel 1, lane 1) and only a band at 37kDa in immunoprecipitations with anti-CDKB1;1 antibody; blotting using anti-CDKB1;1 antibody after immunoprecipitations using anti-CDKA and anti-CDKB1;1 antibodies only recognizes a band in 37kDa (Fig. 1B, panel 2), demonstrating that the anti-CDKB1;1 antibody does not immunoprecipitate CDKA.

### Detection of cyclins D2;2, D4;2, and D5;3 proteins during maize germination

**Fig. 2.**
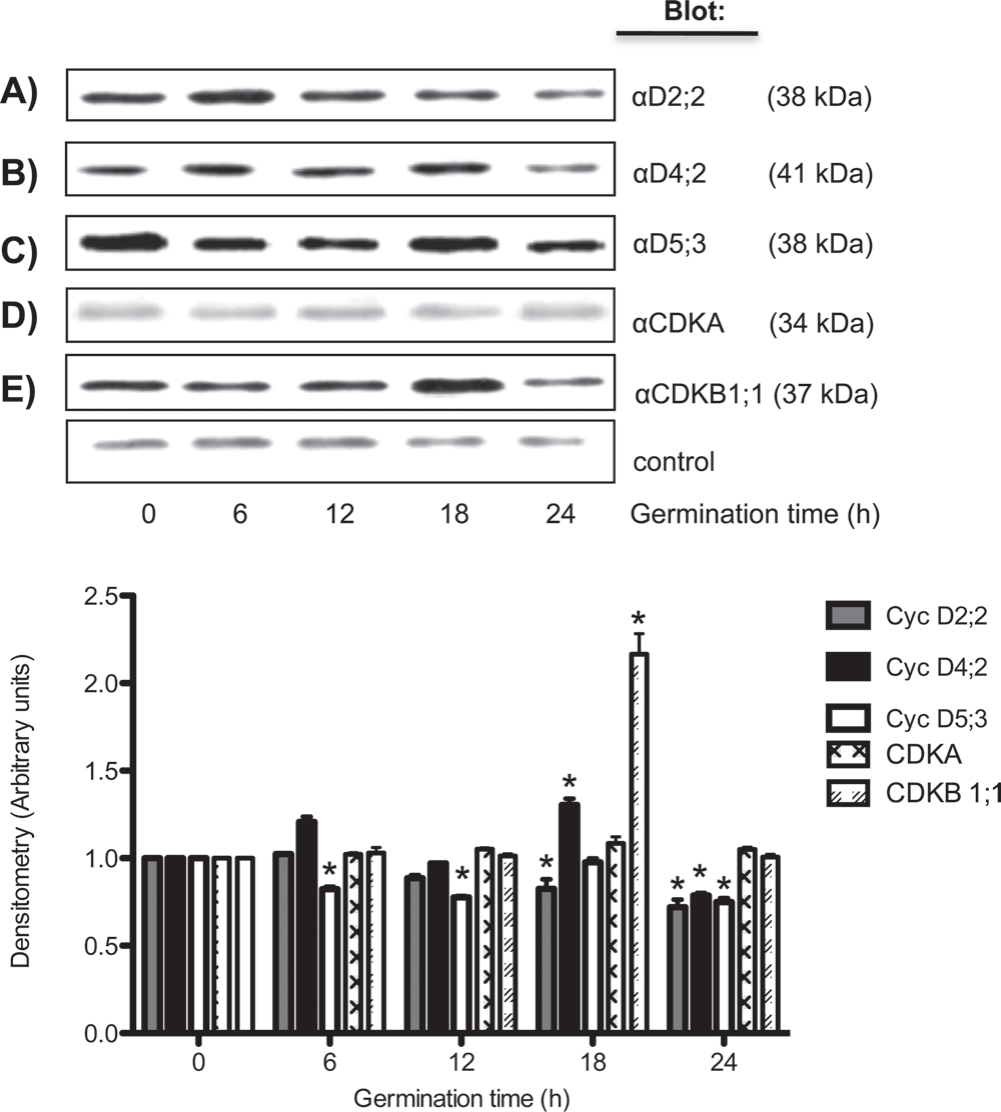
Accumulation of D-type cyclins and CDKs during maize germination. Maize D-type cyclins and CDKs were identified by western blotting using specific antibodies against (A) CycD2;2, (B) CycD4;2, (C) CycD5;3, (D) CDKA, or (E) CDKB1;1 at 0, 6, 12, 18, and 24h after germination. Densitometry analysis was performed relating band intensity of all samples to intensity of the loading control (a 41kDa maize protein recognized by an anti-human cyclin B antibody that is not related to maize B-type cyclin; Lara-Núñez *et al.*, 2008) and then to the dry seed band. Each bar represents the mean±SE from four independent experiments. *Statistically significant value (*P*<0.001) compared to control.

Maize cells contain 17 genes that encode D-type cyclins, and 15 of them are expressed during germination (Buendía-Monreal *et al.*, 2011). To study the expression at the protein level, and their function during maize germination, antibodies against CycD4;2 or against CycD5;3 were used; the anti-CycD4;2 antibody recognized a 41kDa protein that was present in embryo axes of dry seeds and levels peaked at 18h of germination, to be reduced to about half that value at 24h (Fig. 2B). In contrast, the antibody against CycD5;3 detected a 38kDa protein that was also present in dry seeds and protein levels seemed to decrease slightly during germination (Fig. 2C). The behaviour of CycD2;2 has been reported on before (Gutiérrez *et al.*, 2005) and it is very similar to that shown here (Fig. 2A). On the other hand, anti-CDKA antibodies detected a band at 34kDa and levels did not seem to vary much (Fig. 2D), whereas anti-CDKB1;1 antibodies showed a 37kDa protein that varied little in the first hours of germination, reaching a peak at 18h to decrease by 24h of germination (Fig. 2E).

### Stability of D-type cyclins during maize germination

**Fig. 3.**
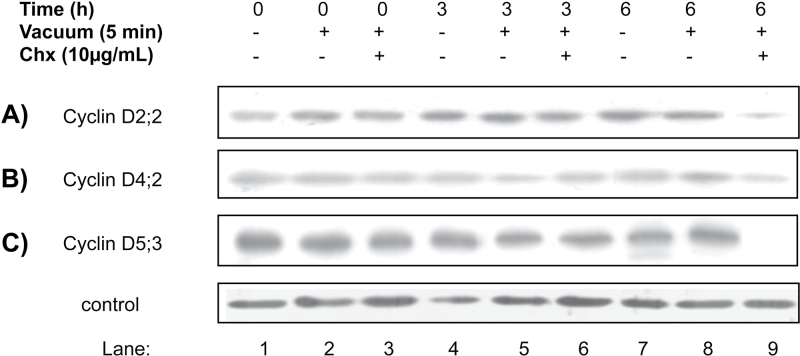
Stability of D-type cyclins during germination. Maize embryo axes were imbibed for 0–6h in the presence of cycloheximide (Chx; introduced by means of vacuum) and then the presence of D-type cyclins was followed by western blot. Lanes 1, 4, and 7, protein extracts from 0, 3, and 6 h-imbibed maize axes in the absence of cycloheximide. Lanes 2, 5, and 8, protein extracts from 0, 3, and 6 h-imbibed maize axes with a 5min vacuum treatment at the beginning of the imbibition time. Lanes 3, 6, and 9, protein extracts from 0, 3, and 6 h-imbibed maize axes treated with vacuum and cycloheximide. Loading control as in Fig. 2.

By definition, cyclins are unstable proteins; the presence at relatively constant levels of maize D-type cyclins during germination was intriguing and therefore their stability was studied. For this purpose, cycloheximide was added to germinating maize axes and the levels of each cyclin at 0, 3, and 6h of imbibition were followed. The levels of the three D-type cyclins importantly decreased by 6h of germination (CycD5;3 could not even be detected; Fig. 3C). As there was still a residual amount of CycD2;2 and CycD4;2 the experiment was prolonged to 9h of germination; by this time no cyclin was detected if cycloheximide was added (Fig. S3). This result indicates that the three cyclins are targeted for degradation during early germination times and, thus, proteins observed in Fig. 2 must be the result of *de novo* synthesis, suggesting a balanced process of synthesis and degradation during maize germination.

### Association of CycD2;2, CycD4;2, and CycD5;3 with CDKs during germination

**Fig. 4.**
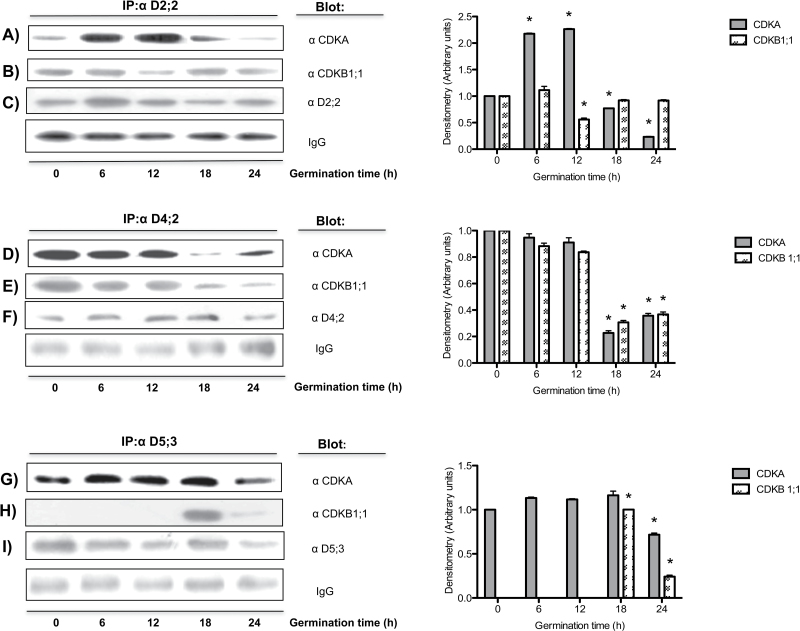
Interaction of D-type cyclins with CDKs during maize germination. Antibodies against cyclins D2;2, 4;2, and 5;3 were used for immunoprecipitation and identification of the associated CDK in protein extracts from 0, 6, 12, 18, and 24 h-germinated axes. (A, B) Co-immunoprecipitation of CycD2;2 with CDKA and CDKB1;1 respectively; (D, E) co-immunoprecipitation of CycD4;2 with CDKA and CDKB1;1 respectively; (G, H) co-immunoprecipitation of CycD5;3 with CDKA and CDKB1;1 respectively. In (H) the intensity of the band at 18h was given a value of 1 as it could not be referred to the null value at time 0; thus, the band at 24h was compared to that at 18h. (C, F, I) Target proteins of the corresponding immunoprecipitating antibodies. Heavy chain IgGs were used as a loading control. Densitometry analysis was performed relating band intensity of all samples to the intensity of the loading control and then to the dry seed band. Each bar represents the mean±SE from three independent experiments. *Statistically significant value (*P*<0.001) compared to control.

Cyclins complexed with CDKs allow the latter to develop kinase activity. Antibodies were used to follow the interaction of the different D-type cyclins with CDKs using immunoprecipitation experiments. The three D-type cyclins interacted with both CDKs (Fig. 4). CycD2;2 had a peak of interaction with CDKA at 12h of germination, strongly decreasing thereafter (Fig. 4A). On the other hand, CycD2;2 seemed to interact equally well at all times with CDKB1;1, with the only exception of the 12h of germination time point, in which association was reduced (Fig. 4B).

Association of CycD4;2 with both CDKA and CDKB1;1 was very similar, as levels between 0 and 12h of germination changed little and then interaction drastically decreased afterwards (Fig. 4D, E).

A very contrasting behaviour was found in the association of CycD5;3 with CDKs; whereas association with CDKA varied little during germination (Fig. 4F), with CDKB1;1 association only took place at 18 and 24h of germination (Fig. 4G).

### Separation of D-type cyclin–CDKA and D-type cyclin–CDKB1;1 complexes

**Fig. 5.**
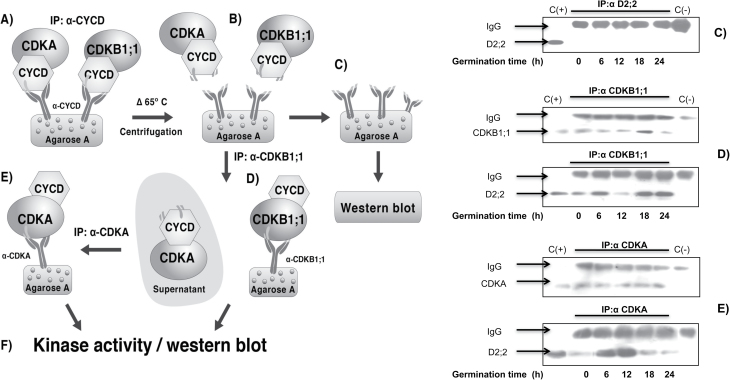
Method of sequential immunoprecipitation. Left-hand panel: (A) immunoprecipitation of D-type cyclins, (B) incubation of immunoprecipitates at 65 °C for 3h and centrifugation, (C) D-type cyclin–CDK complex removal, (D) immunoprecipitation with anti-CDKB1;1 antibody, (E) immunoprecipitation of resulting supernatant after step (C) with anti-CDKA antibody, and (F) kinase activity in immunoprecipitates. Right-hand panel: detection of target proteins after each immunoprecipitation. (C) Recognition of CycD2;2 in immunoprecipitates with anti-CycD2;2 antibody, (D) recognition of CycD2;2 and CDKB1;1 in immunoprecipitates using anti-CDKB1;1 antibody, and (E) recognition of CycD2;2 and CDKA in immunoprecipitates using the anti-CDKA antibody. C(+), Protein extract from 12 h-imbibed maize axes; C(−), protein A–agarose+antibody (no protein extract).

The antibody against CDKA was prepared using the whole CDKA protein and recognized CDKB1;1, although poorly, when protein concentration was above 200 µg (Fig. 1B and Fig. S2D, F); on the other hand, the antibody against CDKB1;1 was prepared using the N-terminal region, which is specific for this protein and therefore only recognizes CDKB1;1. Thus, a strategy was developed to separate D-type cyclins binding to one or the other CDK. As a first step, D-type cyclins were immunoprecipitated using the corresponding antibody (Fig. 5A). Then the immunoprecipitate was heated at 65 °C for 3h to separate the D-type cyclin–CDK complex from protein A–agarose, denaturing the light and heavy chains; after spinning, the supernatant contained the pool of D-type cyclin–CDK complexes. By incubating with protein A–agarose and the anti-CDKB1;1 antibody (Fig. 5D), specific D-type cyclin–CDKB1;1 complexes were obtained in the immunoprecipitate, whereas the remaining supernatant should contain D-type cyclin–CDKA complexes that could be immunoprecipitated with anti-CDKA antibodies (Fig. 5E). Results with CycD2;2 are shown as an example of the reliability of this technique. CycD2;2 is recognized only in protein extracts, but not in immunoprecipitates when complexes have been removed (Fig. 5C, left-hand panel); CycD2;2 and CDKB1;1 are recognized when complexes are immunoprecipitated with anti-CDKB1;1 antibody (Fig. 5D, left-hand panel) and CycD2;2 and CDKA are recognized when the anti-CDKA antibody is used (Fig. 5E, left-hand panel). Assays for CycD4;2 and CycD5;3 are shown in Figure S4. The immunoprecipitate using the anti-CDKB1;1 antibody showed the presence of CDKB1;1 at all germination times (with a peak at 18h), whereas the pattern of association with D-type cyclins was very similar to that shown in Figures 4B, E, and H. Immunoprecipitates with anti-CDKA antibody also showed the presence of CDKA at all germination times and an association pattern with D-type cyclins very similar to that shown in Figs 4A, D, and G.

### Differential kinase activity in D-type cyclin–CDKA and D-type cyclin–CDKB1;1 complexes during maize germination

**Fig. 6.**
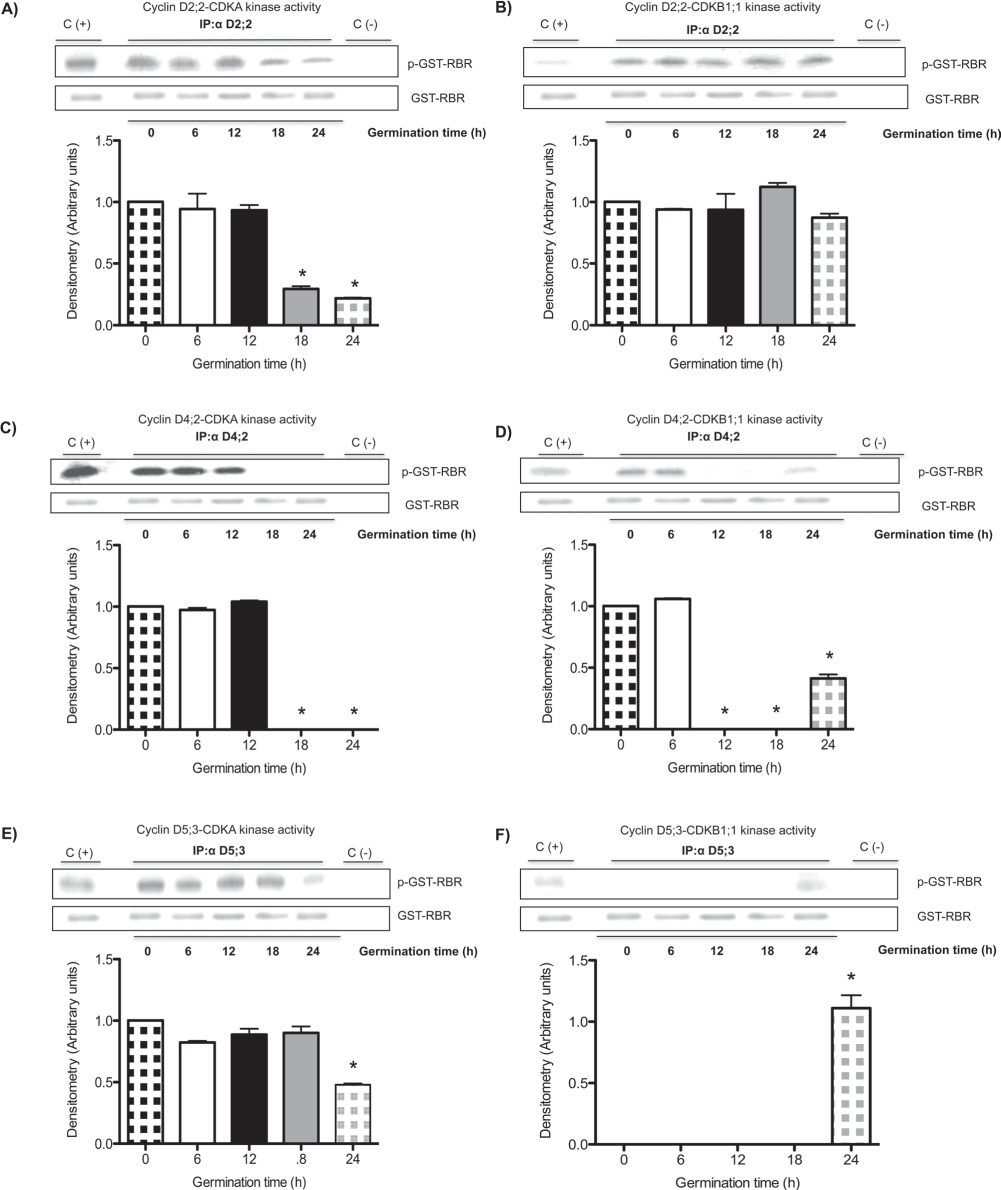
Protein kinase activity in CDKA– or CDKB1;1–D-type cyclin complexes during maize germination. Activity was measured in CDKA–D-type cyclins or CDKB1;1–D-type cyclins complexes at 0, 6, 12, 18, and 24h of germination. (A, B) Kinase activity corresponding to CycD2;2 associated with CDKA or CDKB1,1 respectively. (C, D) Kinase activity corresponding to CycD4;2 associated with CDKA or CDKB1,1 respectively. (E, F) Kinase activity corresponding to CycD5;3 associated with CDKA or CDKB1,1 respectively. Again, in (F), the intensity of the band at 24h was taken as 1 as it could not be referred to the null value at time 0. The amount of GST–RBR protein (37kDa) added as a substrate for kinase activity was used as the loading control. Densitometry analysis was performed relating band intensity of all samples to intensity of the loading control and then to the dry seed band. C(+), Kinase activity in cyclin–CDK complexes pulled-down by rice CKS protein; C(−), D-type cyclin–CDK complexes without RBR added. Each bar represents the mean±SE from three independent experiments. *Statistically significant value (*P*<0.001) compared to control.

Using the conditions established above, D-type cyclin–CDK complexes were separated and kinase activity onto pRBR was measured in every protein complex. Kinase activity in the CycD2;2–CDKA complex did not change between 0 and 12h of germination and then gradually decreased (Fig. 6A). However, there was no variation in kinase activity in the CycD2;2–CDKB1;1 complex during germination (Fig. 6B). In the complex formed by CycD4;2 and CDKA, again no variation in kinase activity was detected between 0 and 12h, but after that time activity could not be detected (Fig. 6C); when complexed with CDKB1;1 kinase activity was detected at 0 and 6h of germination, after which activity was not detectable until 24h of germination (Fig. 6D).

In the CycD5;3–CDKA complex, kinase activity was similar between 0 and 18h of germination and then decreased by 75% (Fig. 6E), whereas activity when containing CDKB1;1 was not detected in the 0–18h period (Fig. 6F). Kinase activity was only detected at 24h of germination.

As a positive control for D-type cyclin–CDK kinase activity, use was made of proteins pulled-down by the rice CKS protein (our unpublished data), an orthologue of yeast p13Suc1, which strongly binds Cdc2-type CDKs (Ducommun *et al.*, 1991).

**Fig. 7.**
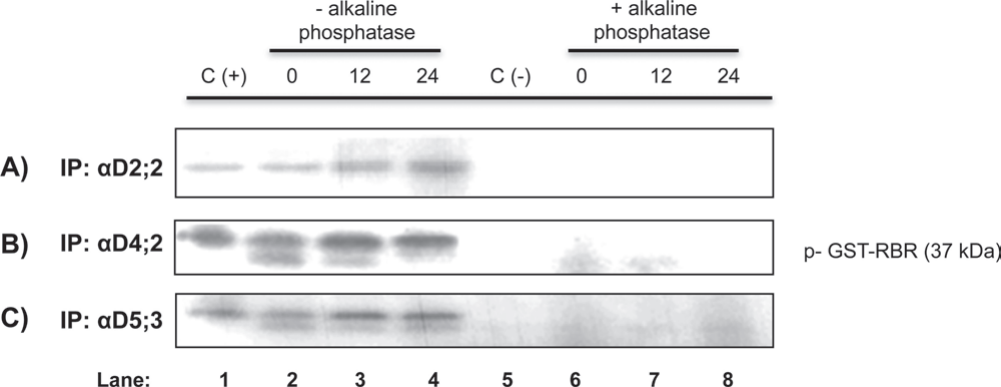
Effect of alkaline phosphatase in kinase activity. (A) Anti-CycD2;2 immunoprecipitates of axes imbibed for 0, 12, and 24h with or without alkaline phosphatase; (B) same as (A) but using the anti-CycD4;2 antibody; (C) same as (A) but using the anti-CycD5;3 antibody.

Kinase activity in D-type cyclin–CDK complexes is dependent upon prior activating phosphorylation at a specific residue, Thr–160 (or the equivalent in every CDK; Fig. S1B). To test whether active D-type cyclin–CDK complexes contained activating phosphorylated residues, the three anti-D-type cyclin antibodies were used to immunoprecipitate complexes and these were treated or not with alkaline phosphatase prior to performing the kinase assay. The presence of a phosphatase totally eliminated kinase activity in all complexes, at every germination time tested, strongly suggesting that these complexes depend on a prior phosphorylation step to function (Fig. 7 and Fig. S5).

## Discussion

The cell cycle is reactivated when seed germination begins. It has been reported that about 80% of cells in the dry seed are in G_1_ phase (Bewley and Black, 1994), and it is in this phase that D-type cyclins should play an important role in cell cycle activation and progression. The three D-type cyclin proteins studied here—D2;2, D4;2, and D5;3—are present in dry seeds and during germination with apparently minimal fluctuation, particularly in the early stages of germination; however, the assays using cycloheximide show that these three proteins are unstable during these early hours; therefore, cyclin levels observed must be the result of a balanced process of synthesis and degradation that takes place during maize germination. It is difficult to know whether both the ‘stored’ and the newly synthesized D-type cyclins form useful, functional complexes with CDKs, even though it is clear that active D-type cyclin–CDK complexes are readily detectable at times when visible cyclin decay is evident, after 3–6h of germination. It could be speculated that the active early germination ‘stored’ complexes would work to allow the accumulation of new complexes that then would take the cell cycle, and the germination process, forward. There is, however, no proof of the existence of such a cascade regulation or if the active, ‘stored’ complexes are located in physiologically relevant places so that they could help switch on the cell cycle.

All three cyclins associate with both CDKA and CDKB1;1 but show variations in association timing. Cyclins D2;2, D4;2, and D5;3 form complexes with CDKA at all times, and association is reduced by 24h of germination, when mitosis is starting in meristem cells (Baiza *et al.*, 1989). Complexes of cyclins with CDKB1;1 show differences, but the most interesting is the lack of association with CycD5;3 during the early germination times, only to appear later; it should be recalled that the two CDKs are present throughout the germination process (Fig. 2). As will be discussed below, this differential association will necessarily be reflected in kinase activity. Since it had been reported that only a few plant D-type cyclins interacted with B-type CDKs (Swaminathan *et al.*, 2000), it becomes remarkable that all D-type cyclins studied here show interaction. Incidentally, it should be stated that maize cells appear to contain three almost identical A-type CDKs, and that our antibodies cannot distinguish among them. Thus, regarding A-type CDKs the results reported here must be the sum of the associations and activity of the different possible D-type cyclin–CDKA complexes. There was no way we could discriminate between them.

Measuring total kinase activity in immunoprecipitates using every D-type cyclin antibody gave results that were difficult to interpret (results not shown). However, when complexes of the different cyclins with every CDK were separated by sequential immunoprecipitation, the results were revealing. As explained above, a sequential immunoprecipitation technique was applied by which an initial immunoprecipitation with anti-D-type cyclin antibodies was followed by a second immunoprecipitation with the anti-CDKB1;1 antibody. Then the remaining supernatant was immunoprecipitated with the anti-CDKA antibody (Fig. 5 and Fig. S4). These sequential immunoprecipitations showed that kinase activity in CycD2;2–CDKA complexes compared well with the amount of the corresponding complexes found at every germination time measured; but interestingly, that active CycD2;2–CDKB1;1 complexes were present throughout germination, a result that suggests roles for D-type cyclins not only in the G_1_ phase, but also in G_2_–M, when B-type CDKs should be active. Evidence for D-type cyclins participating in G_2_–M processes in plants has been accumulating (Mészáros *et al.*, 2000).

Activity in CycD4;2–CDKs complexes showed some differences to that shown by CycD2;2 complexes; with CDKA there was activity up to 12h of germination and then activity could not be detected, in close agreement with the association capacity; this result may indicate that CycD4;2–CDKA is important for the G_1_–S transition. On the other hand, with CDKB1;1 there was early activity and then no activity at all between 12 and 18h of germination, although it reappeared at 24h. This result may indicate that complex formation is not enough, and the possibility of regulation of kinase activity by either CDK phosphorylation status or the association with inhibitory proteins such as KIP1-related proteins (KRPs) should also be considered.

Again, CycD5;3 association with CDKs gave the more contrasting result. Whereas, when associated with CDKA there was similar kinase activity between 0 and 18h of germination and then activity declined, following the pattern of association, exactly the opposite was observed with CDKB1;1: no activity at all was detected in the 0–18h period and then the complex was formed, by 18h, and activated at 24h, prior to the onset of the M phase (Baiza *et al.*, 1989). This would be the expected result as no association of CycD5;3 with CDKB1;1 was detected during early stages of germination. Apparently, with CDKA CycD5;3 would form a G_1_-S kinase and with CDKB1;1 it would form a G_2_-M kinase.

As indicated above, in embryos of dry seeds of different species, cells in meristematic tissues, the only cell populations that proliferate, have mostly a G_1_-phase DNA content (Bewley and Black, 1994) and this is the case for maize. Although there is no absolute synchrony in cell populations, evidence indicates that by 24h of germination cells in maize embryo axes are already in G_2_ phase and by 28h cells are getting into the M phase (Baiza *et al.*, 1989). Thus, maize D-type cyclins would appear to function with, and regulate G_1_-S and G_2_-M CDKs and these results may indicate a non-redundant but differential physiological role for every D-type cyclin–CDK complex. However, much is still ignored about the time of action, activation, associations, and location of the different D-type cyclins and CDKs in plants. The study of their location will be an important topic to follow. Also, the phosphorylation status of each CDK when bound to a cyclin could determine whether a complex is active or inhibited.

In conclusion, during maize germination the temporal differences in formation and activation of complexes integrated by different D-type cyclins and CDKs suggest partially non-redundant behaviour, indicating that every complex has some specific role to play during the first cell cycles necessary for a seed to germinate and become a seedling.

## Supplementary material

Supplementary material is available at *JXB* online.

Supplementary Fig. S1. Comparison of maize D-type cyclin sequences. (A) Alignment of carboxyl ends of the 17 maize D-type cyclins and percentage identity of Cyclins D4;2 and D5;3 compared to all maize cyclins. (B) Comparison of maize CDK sequences. Motif 1, sequence used for production of anti-CDKB1;1 antibodies; motif 2, canonical PSTAIRE-cyclin binding sequence (in Cdc2-type kinases like CDKA), PPTAL(M)RE in CDKB. (*) Represents phosphorylatable T14, Y15, and T160 residues, conserved in all CDKs.

Supplementary Fig. S2. Validation of antibodies against D-type cyclins and CDKs. (A, B) Lanes 1 and 3, protein extracts from non-imbibed maize axes and recombinant proteins incubated with the corresponding antibody; lanes 2, recombinant proteins incubated only with pre-immune serum. (A) Antibodies against GST–CycD4;2 and GST–CyclinD5;3; (B) antibodies against His-CDKA and GST–CDKB1;1. (C, D) Specificity of antibodies. (C) Lanes 1, protein extracts from non-imbibed maize axes; lanes 2, GST–CycD4;2; lanes 3, GST–CycD5;3; (C1) western blot using anti-CycD4;2 antibody; (C2) western blot using anti-CycD5;3 antibody. (D) Lanes 1, protein extracts from non-imbibed maize axes; lanes 2, His-CDKA; lanes 3, GST–CDKB1;1; (D1) western blot using anti-CDKA antibody; (D2) western blot using anti-CDKB1;1 antibody. (E, F) Recognition of CDKs. (E1) Increasing concentrations of protein extracts (50–300 μg) from non-imbibed maize axes and recognition of CDKs using the anti-CDKA antibody. Notice the recognition of a 37kDa band (CDKB1;1) at 250 μg of protein; (E2) same as above but recognition with anti-CDKB1;1 antibody (only CDKB1;1 is recognized). Membranes stained with Ponceau red are shown as the loading control. (F) Immunoprecipitation with anti-CDKA antibody. (F1) Immunoprecipitation of samples with increasing concentrations of protein extracts (100–300 μg) from non-imbibed maize axes, using anti-CDKA antibody and recognition by anti-CDKA antibodies of 34kDa (CDKA) and 37kDa (CDKB1;1) bands; the latter is observed only in the 300 μg sample. (F2) Western blot using anti-CDKB1;1 antibody of proteins immunoprecipitated by anti-CDKA antibodies in samples of increasing concentrations of protein extracts from non-imbibed maize axes (150, 200, and 300 µg). Only the 37kDa band is detected at 300 µg. C(+), Proteins extracts from non-imbibed axes (50 µg); C(−), immunoprecipitation with anti-CDKA antibody, no protein extract added.

Supplementary Fig. S3. Stability of D-type cyclins during germination. Maize embryo axes were imbibed for 9h in the presence of cycloheximide (introduced by means of a vacuum) and then the presence of D-type cyclins was followed by western blot. Lanes 1, 4, and 7, protein extracts from 9 h-imbibed maize axes in the absence of cycloheximide. Lanes 2, 5, and 8, protein extracts from 9 h-imbibed maize axes with a 5min vacuum treatment at the beginning of imbibition. Lane 3, 6, and 9, protein extracts from 9 h-imbibed maize axes treated with vacuum and cycloheximide. Loading control as in Fig. 2.

Supplementary Fig. S4. Validation of the sequential immunoprecipitation technique (according to Fig. 5). Lane C: immunoprecipitation (0, 6, 12, 18, and 24h of germination) and western blot of each D-type cyclin after heat treatment (65 °C, 3h) and removal of D-type cyclin–CDK complexes. Lane D: immunoprecipitation of CDKB1;1 from the supernatant containing D-type cyclin–CDK complexes from step B and identification of CDKB1;1 and D-type cyclins by western blot. Lane E: immunoprecipitation of CDKA from the supernatant obtained in step D and identification of CDKA and D-type cyclins by western blot. Positive control, identification of the target protein in protein extracts from non-germinated seed axes. Negative control, high-molecular-weight IgGs.

Supplementary Fig. S5. Alkaline phosphatase and CDK activity. Lane 1 (C+), kinase activity in cyclin–CDK complexes pulled down by CKS protein; lane 2, kinase activity in anti-CycD2;2 immunoprecipitate; lane 3, dephosphorylation of substrate used in lane 2 (RBR protein) by alkaline phosphatase; lane 4, inhibition of alkaline phosphatase activity by 40min pre-incubation with inhibitor and kinase activity in anti-CycD2;2 immunoprecipitate; lane 5 C(−), anti-CycD2;2 immunoprecipitate with no protein extract added; lane 6, pre-incubation of alkaline phosphatase with inhibitor (40min), then substrate and ^32^P were added and then incubation with anti-CycD2;2 immunoprecipitate; lane 7, pre-incubation of alkaline phosphatase with ^32^P and inhibitor, then the substrate and finally anti-CycD2;2 immunoprecipitate; lane 8, preincubation of alkaline phosphatase and ^32^P (40min), then the kinase assay.

Supplementary Fig. S6. Kinase activity after a high-temperature treatment. (A) Lane 1 (C+), kinase activity in cyclin–CDK complexes bound to CKS protein; lane 2, kinase activity in CycD2;2–CDKA complexes (heat-treated); lane 3, kinase activity in CycD2;2–CDKB1;1 complexes (heat-treated); lane 4, kinase activity in anti-CDKA immunoprecipitates (no heat treatment); lane 5, kinase activity in anti-CDKB1;1 immunoprecipitates (no heat treatment); lane 6 (C−), kinase activity in anti-CycD2;2 immunoprecipitates with no protein extract added. (B) Lane 1 (C+), kinase activity in cyclin–CDK complexes bound to CKS protein; lane 2, kinase activity in anti-CycD2;2 immunoprecipitates; lane 3, kinase activity in anti-CDKA immunoprecipitates; lane 4, kinase activity in anti-CDKB1;1 immunoprecipitates; lane 5, kinase activity in cyclin–CDK complexes pulled down by a p13Suc1 resin; lane 6, kinase activity in cyclin–CDK complexes pulled down by a p13Suc1 resin treated at 65 °C for 3h; lane 7, kinase activity in anti-CycD2;2 immunoprecipitates with no protein extract added.

Supplementary Data

## Abbreviations:

CDK: cyclin-dependent kinase
Cyc: cyclin
GST: glutathione S-transferase
RBR: retinoblastoma-related.
