# Supplementary material for: Complexes of D-type cyclins with CDKs during maize germination
Source: J Exp Bot. 2013 Oct 14;64(18):5661–71. doi: 10.1093/jxb/ert340 (PMC3871821; doi:10.1093/jxb/ert340)
Supplement: Supplementary Data [file supp_64_18_5661__index.html]

Complexes of D-type cyclins with CDKs during maize germination — Complexes of D-type cyclins with CDKs during maize germination — Supplementary Data 

# Complexes of D-type cyclins with CDKs during maize germination

## Supplementary Data

Data files

**Files in this Data Supplement:**

- Supplementary Data - Supplementary Data
