# Supplementary material for: Complexes of D-type cyclins with CDKs during maize germination
Source: J Exp Bot. 2013 Oct 14;64(18):5661–71. doi: 10.1093/jxb/ert340 (PMC3871821; doi:10.1093/jxb/ert340)
Supplement: Supplementary Data [file supp_ert340_jexbot109082_file001.pdf]

COMPLEXES OF D-TYPE CYCLINS WITH CDKS DURING MAIZE GERMINATION

Silvia K Godínez-Palma[1] , Elpidio García[1] , María de La Paz Sánchez[2] , Fernando Rosas[1], Jorge M Vázquez-Ramos[1]

A)

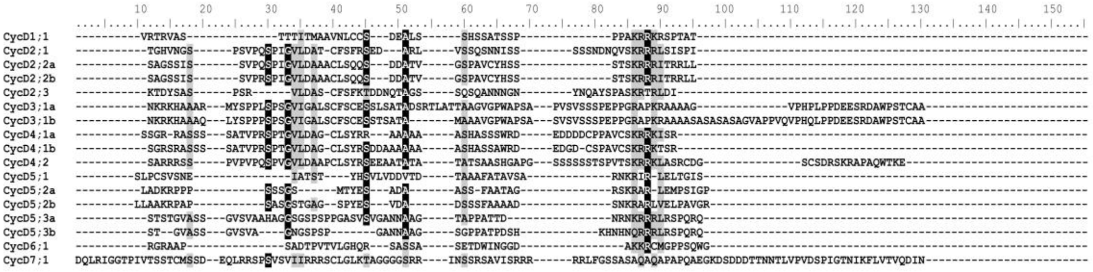

	CycD1;1	CycD2;1	**CycD2;2a**	**CycD2;2b**	CycD2;3	CycD3;1a	CycD3;1b	CycD4;1a	CycD4;1b	**CycD4;2**	CycD5;1	CycD5;2a	CycD5;2b	**CycD5;3a**	**CycD5;3b**	CycD6;1	CycD7;1
CycD4;2	12.30%	28.40%	**30.60%**	**29.50%**	17.00%	21.00%	19.40%	30.70%	32.20%	**ID**	8.80%	12.20%	13.10%	**17.70%**	**13.30%**	7.80%	6.60%
CycD5;3a	10.90%	14.40%	**17.40%**	**17.40%**	13.00%	7.00%	5.50%	13.60%	16.90%	**17.70%**	12.10%	10.70%	10.60%	**ID**	**64.60%**	6.20%	5.00%
CycD5;3b	8.00%	13.00%	**15.60%**	**15.60%**	13.80%	5.00%	4.60%	12.60%	14.00%	**13.30%**	3.20%	7.80%	6.10%	**64.60%**	**ID**	6.50%	4.20%

B)

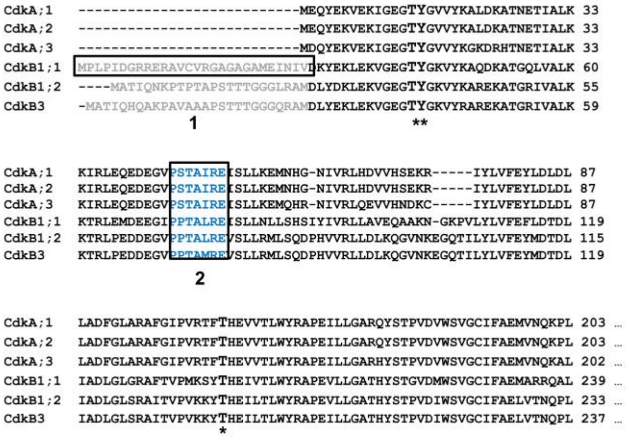

53.7% identity between CDKAs and CDKB1;1

FIGURE S1

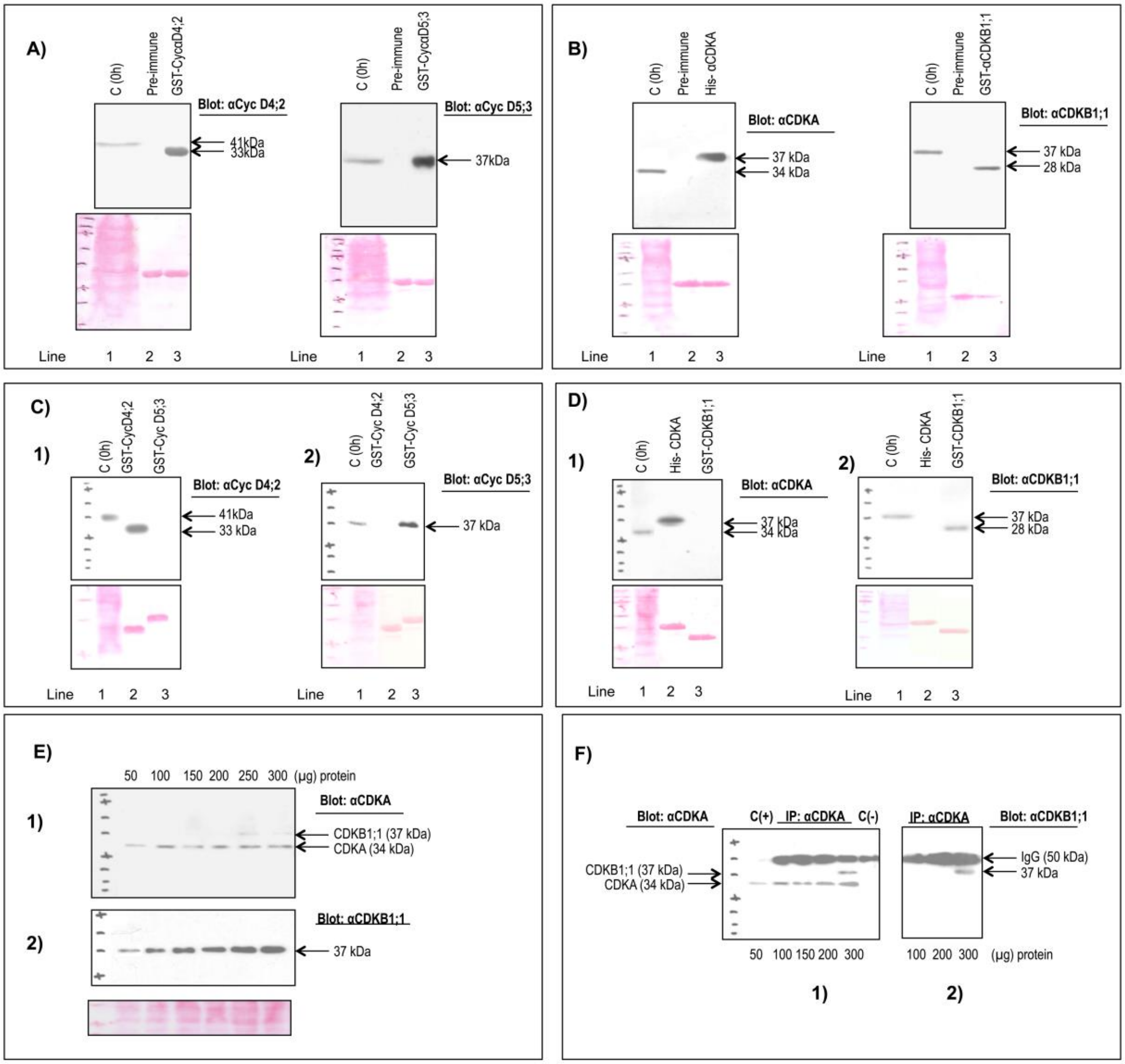
A)
C (0h)
Pre-immune
GST-CycαD4;2
Blot: αCyc D4;2
41kDa
33kDa
GST-CycαD5;3
Blot: αCyc D5;3
37kDa
Line 1 2 3
B)
His- αCDKA
Blot: αCDKA
37 kDa
34 kDa
GST-αCDKB1;1
Blot: αCDKB1;1
28 kDa
C)
1)
GST-CycD4;2
GST-Cyc D5;3
2)
GST-Cyc D4;2
D)
His- CDKA
GST-CDKB1;1
E)
50 100 150 200 250 300 (μg) protein
Blot: αCDKA
CDKB1;1 (37 kDa)
CDKA (34 kDa)
Blot: αCDKB1;1
F)
C(+)
IP: αCDKA
C(-)
IgG (50 kDa)
50 100 150 200 300
100 200 300 (μg) protein

FIGURE S2

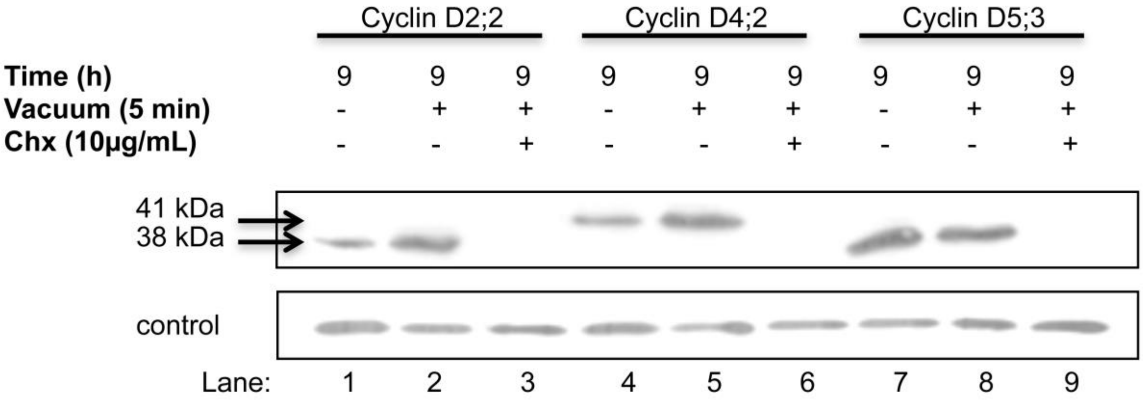
Cyclin D2;2
Cyclin D4;2
Cyclin D5;3
Time (h)
9 9 9 9 9 9 9 9 9
Vacuum (5 min)
- + + - + + - + +
Chx (10µg/mL)
- - + - - + - - +
41 kDa
38 kDa
control
Lane:
1 2 3 4 5 6 7 8 9

FIGURE S3

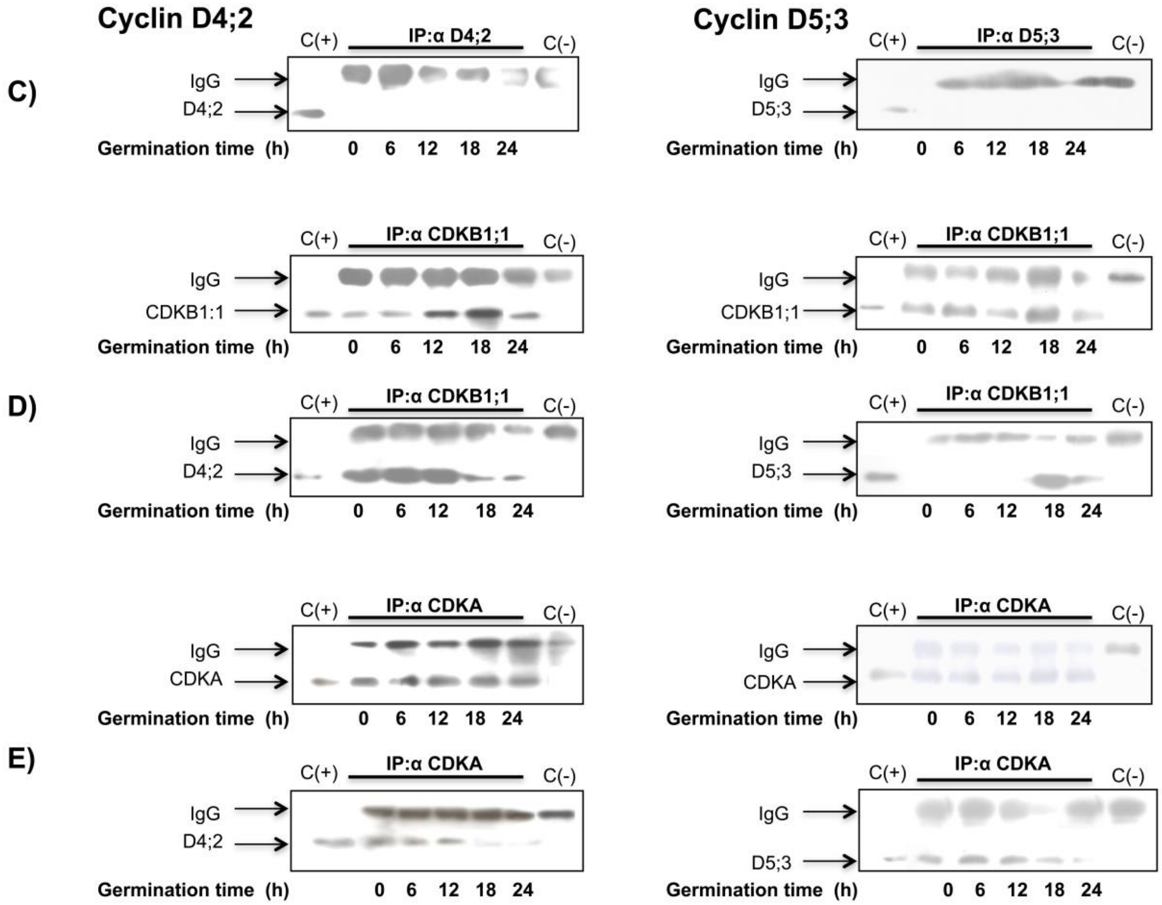

Cyclin D4;2
Cyclin D5;3
C)
D)
E)
C(+)
IP:α D4;2
C(-)
IgG
D4;2
Germination time (h)
0 6 12 18 24
IP:α D5;3
D5;3
IP:α CDKB1;1
CDKB1:1
CDKB1;1
IP:α CDKA
CDKA

FIGURE S4

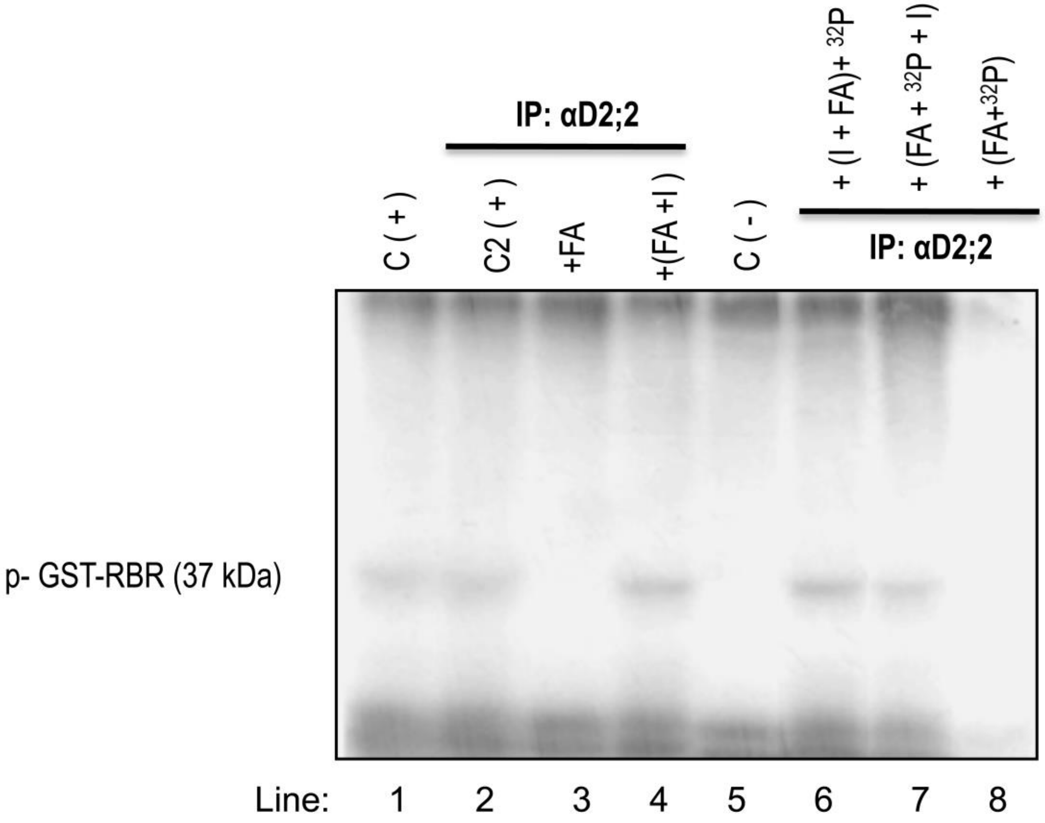
IP: αD2;2
C (+)
C2 (+)
+FA
+(FA +I)
C (-)
+ (I + FA)+ 32P
+ (FA + 32P + I)
+ (FA+32P)
IP: αD2;2
p- GST-RBR (37 kDa)
Line: 1 2 3 4 5 6 7 8

FIGURE S5

A)

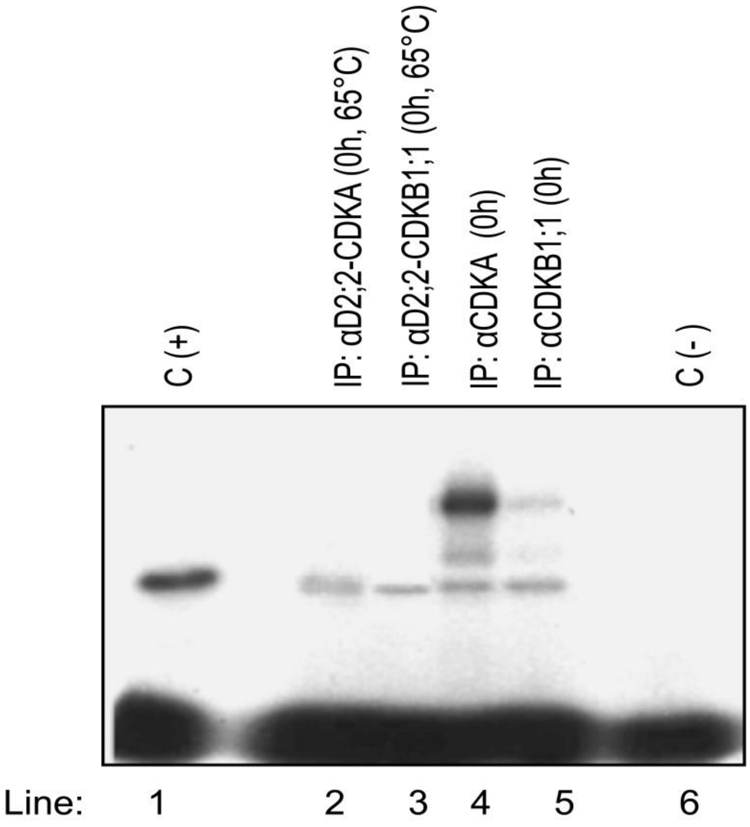
C (+)
IP: αD2;2-CDKA (0h, 65°C)
IP: αD2;2-CDKB1;1 (0h, 65°C)
IP: αCDKA (0h)
IP: αCDKB1;1 (0h)
C (-)
Line: 1 2 3 4 5 6

B)

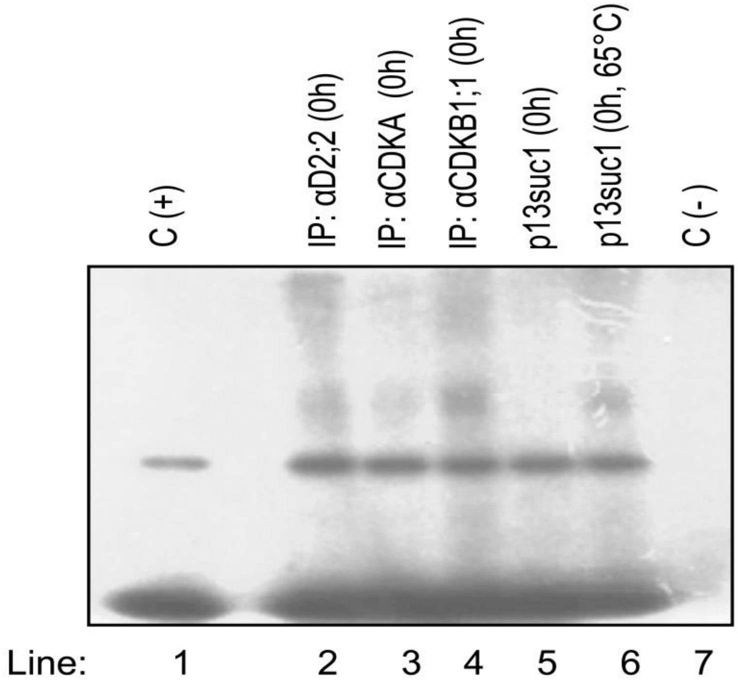
C (+)
IP: αD2;2 (0h)
IP: αCDKA (0h)
IP: αCDKB1;1 (0h)
p13suc1 (0h)
p13suc1 (0h, 65°C)
C (-)
Line: 1 2 3 4 5 6 7

FIGURE S6

Supplementary data.

Supplementary Figure S1. Comparison of maize D-type Cyclins sequences. A) Alignment of carboxyl ends of the 17 maize D-type Cyclins and percentage identity of Cyclins D4;2 and D5;3 compared to all maize cyclins. B) Comparison of maize CDK sequences. Motif 1, sequence used for production of anti-CDKB1;1 antibodies; Motif 2, canonical PSTAIRE-Cyclin binding sequence (in Cdc2-type kinases like CDKA), PPTAL(M)RE in CDKB. (*) Represents phosphorylatable T14, Y15 and T160 residues, conserved in all CDKs.

Supplementary Figure S2. Validation of antibodies against D-type cyclins and CDKs. Panels A and B, lanes 1 and 3, protein extracts from non-imbibed maize axes and recombinant proteins incubated with the corresponding antibody, lanes 2, recombinant proteins incubated only with pre-immune serum. Panel A, antibodies against GST-Cyclin D4;2 and GST-CyclinD5;3; Panel B, antibodies against His-CDKA and GST-CDKB1;1. Panels C and D, Specificity of antibodies. Panel C, lanes 1, protein extracts from non-imbibed maize axes, lanes 2, GST-CycD4;2, lanes 3, GST-CycD5;3; C1), Western blot using anti-CycD4;2 antibody, C2) Western blot using anti-CycD5;3 antibody. Panel D, lanes 1, protein extracts from non-imbibed maize axes, lanes 2, His-CDKA, lanes 3, GST-CDKB1;1; D1), Western blot using anti-CDKA antibody, D2), western blot using anti-CDKB1;1 antibody. Panels E and F, recognition of CDKs. Panel E1, increasing concentrations of protein extracts (50 to 300 µg) from non-imbibed maize axes and recognition of CDKs using the anti-CDKA antibody. Notice the recognition of a 37kDa band (CDKB1;1) at 250 µg protein; panel E2, same as above but recognition with anti-CDKB1;1 antibody (only CDKB1;1 is recognized). Membranes stained with Ponceau Red are shown as loading control. Panel F, immunoprecipitation with anti-CDKA antibody. F1, immunoprecipitation of samples with increasing concentrations of protein extracts (100 to 300 µg) from non-imbibed maize axes, using anti-CDKA antibody and recognition by anti-CDKA antibodies of

34 kDa (CDKA) and 37 kDa (CDKB1;1) bands, the latter is observed only in the 300 µg sample. F2, western blot using anti-CDKB1;1 antibody of proteins immunoprecipitated by anti-CDKA antibodies in samples of increasing concentrations of protein extracts from non-imbibed maize axes (150, 200 y 300 µg). Only the 37 kDa band is detected at 300 µg. C(+), proteins extracts from non-imbibed axes (50 µg); C(-), immunoprecipitation with anti-CDKA antibody, no protein extract added.

Supplementary Figure S3. Stability of Cyclins D during germination. Maize embryo axes were imbibed for 9 h in the presence of cycloheximide (introduced by means of vacuum) and then the presence of Cyclins D was followed by western blot. Lanes 1, 4 and 7, protein extracts from 9 h imbibed maize axes in the absence of cycloheximide. Lanes 2, 5 and 8, protein extracts from 9 h imbibed maize axes, with a 5 min vacuum treatment at the beginning of the imbibition time. Lane 3, 6 and 9, protein extracts from 9 h imbibed maize axes, treated with vacuum and cycloheximide. Loading control as in Fig. 2.

Supplementary Figure S4. Validation of the sequential immunoprecipitation technique (according to Fig. 5). Lane C) Immunoprecipitation (0, 6, 12, 18 and 24h germination) and western blot of each one of D-type Cyclins after heat treatment (65°C, 3h) and removal of D-type Cyclins-CDKs complexes. Lane D) Immunoprecipitation of CDKB1;1 from the supernanat containing D-type Cyclins-CDKs complexes from step B and identification of CDKB1;1 and D-type Cyclins by western blot. Lane E) Immunoprecipitation of CDKA from the supernatant obtained in step D and identification of CDKA and D-type Cyclins by western blot. Positive control, identification of the target protein in protein extracts from non-germinated seed axes. Negative control, high molecular weight IgGs.

Supplementary Figure 5. Alkaline phosphatase and CDK activity. Lane 1 (C+), kinase activity in cyclin-CDK complexes pulled down by CKS protein; lane 2, kinase activity in anti-CycD2;2 immunoprecipitate; lane 3, dephosphorylation of

substrate used in lane 2 (RBR protein) by alkaline phosphatase; lane 4, inhibition of alkaline phosphatase activity by 40 min pre-incubation with inhibitor and kinase activity in anti-CycD2;2 immunoprecipitate; lane 5 C(-), anti-CycD2;2 immunoprecipitate with no protein extract added; lane 6, pre-incubation of alkaline phosphatase with inhibitor (40 min), then substrate and ^{32}P were added and then incubation with anti-CycD2;2 immunoprecipitate; lane 7, pre-incubation of alkaline phosphatase with ^{32}P and inhibitor, then the substrate and finally anti-CycD2;2 immunoprecipitate; lane 8, preincubation of alkaline phosphatase and ^{32}P (40 min), then the kinase assay.

Supplementary Figure 6. Kinase activity after a high temperature treatment. Panel A, lane 1 (C+), kinase activity in cyclin-CDK complexes bound to CKS protein; lane 2, kinase activity in CycD2;2-CDKA complexes (heat-treated); lane 3, kinase activity in CycD2;2-CDKB1;1 complexes (heat-treated); lane 4, kinase activity in anti-CDKA immunoprecipitates (no heat treatment); lane 5, kinase activity in anti-CDKB1;1 immunoprecipitates (no heat treatment); lane 6 (C-), kinase activity in anti-CycD2;2 immunoprecipitates with no protein extract added. Panel B, lane 1 (C+), kinase activity in cyclin-CDK complexes bound to CKS protein, lane 2, kinase activity in anti-CycD2;2 immunoprecipitates; lane 3, kinase activity in anti-CDKA immunoprecipitates; lane 4, kinase activity in anti-CDKB1;1 immunoprecipitates; lane 5, kinase activity in cyclin-CDK complexes pulled down by a p13Suc1 resin; lane 6, kinase activity in cyclin-CDK complexes pulled down by a p13Suc1 resin treated at 65°C for 3h; lane 7, kinase activity in anti-CycD2;2 immunoprecipitates with no protein extract added.
